# Prevalence and Associated Factors of Depressive Symptoms in Patients with Hypertension in Eastern Sudan: A Hospital-Based Cross-Sectional Study

**DOI:** 10.3390/healthcare14213805

**Published:** 2026-11-07

**Authors:** Sami S. Alharthi, Saeed M. Omar, Raghad A. Alotaibi, Hatim Y. Alharbi, Afnan A. Alwabili, Ishag Adam

**Affiliations:** 1 Department of Medicine, College of Medicine, Taif University, Taif 21944, Saudi Arabia; s.s.alharthi@tu.edu.sa; 2 Faculty of Medicine, Gadarif University, Gadarif 32211, Sudan; drsaeedomar@yahoo.com; 3 College of Medicine, Sulaiman Al Rajhi University, Al Bukayriah 51941, Saudi Arabia; 211210179@srcolleges.org; 4 Department of Psychiatry, College of Medicine, Qassim University, Buraydah 52389, Saudi Arabia; hy.alharbi@qu.edu.sa (H.Y.A.); a.alwabili@qu.edu.sa (A.A.A.); 5 Department of Obstetrics and Gynecology, College of Medicine, Qassim University, Buraydah 52389, Saudi Arabia

**Keywords:** prevalence, depression, associated factors, hypertension, age, tobacco use

## Abstract

Background: Hypertension poses a significant global health threat and is associated with various mental health conditions, including depression. There are no published data on the prevalence of depressive symptoms in patients with hypertension in Sudan. Thus, this study aimed to assess the prevalence of depressive symptoms in patients with hypertension in Eastern Sudan and identify the associated factors. Methods: This hospital-based cross-sectional study was conducted in Gadarif, Eastern Sudan. Data on demographic characteristics, anthropometrics, and clinical history were obtained. Depressive symptoms were assessed using the Patient Health Questionnaire (PHQ-9), and a multivariate binary analysis was performed. Results: A total of 474 adults with hypertension were enrolled in this study: 162 (34.2%) males and 312 (65.8%) females. The median (interquartile range, IQR) age was 53.0 (45.0–60.0) years. The median (IQR) duration of hypertension was 5.0 (3.0–7.0) years. Of the participants, 42 (8.9%, 95% CI = 6.5–11.7%) had depressive symptoms. The logistic regression analysis showed that increasing age (adjusted odds ratio, AOR = 1.05, 95% confidence interval, CI = 1.01–1.10), being unmarried (AOR = 4.27, 95%, CI = 1.71–10.67), and tobacco use (AOR = 2.45, 95% CI = 1.04–5.80) were associated with depressive symptoms. Sex, the duration of hypertension, the number of medications, education level, and comorbidities were not associated with depressive symptoms. Conclusions: This study revealed that, in patients with hypertension, 8.9% had depressive symptoms, which were significantly associated with older age, being unmarried, and tobacco use. These findings emphasize the need for targeted mental-health interventions that address social and lifestyle factors to improve the overall well-being of hypertensive patients.

## 1. Introduction

Hypertension is a prevalent chronic condition affecting approximately 1.13 billion people globally and is recognized as a significant public health challenge [1]. The prevalence of hypertension is increasing, especially in low- and middle-income countries, where the increasing rate of hypertension is further aggravated by urbanization, lifestyle changes, and restricted access to healthcare [2,3]. In Sub-Saharan Africa, the prevalence of hypertension has risen significantly, reaching 48% among women and 34% among men in 2019 [4]. This could be explained by how poorly controlled hypertension is among patients [5,6]. For example, in Sudan, the prevalence of hypertension has been steadily increasing, with estimates suggesting that approximately 40.8% of adults are affected and that most patients have not adhered to their medications [6,7]. The presence of hypertension is often associated with various comorbidities, including mental health disorders such as depression [8,9,10,11,12]. Previous studies indicated that individuals with hypertension are at a higher risk of experiencing depression, which can adversely impact their overall health and complicate the management of hypertension [8,9,10,11,12]. Moreover, depression among hypertensive patients can lead to poorer health outcomes, including increased comorbidity and mortality rates. Depression is characterized by a range of symptoms, including persistent sadness, loss of interest in activities, and cognitive impairments, that can hinder adherence to the treatment regimens and lifestyle modifications necessary for managing hypertension [13]. The bidirectional relationship between hypertension and depression highlights the importance of understanding the prevalence of depression in this population and identifying associated factors. Despite the growing recognition of the interplay between hypertension and depression, no data exist on this issue in Sudan, particularly in the eastern regions of the country. On a wider scale, a high prevalence of depression was reported among hypertensive African patients, such as those in Ethiopia, where the rate of depression is 32.4% [8]; Morocco, where the rate is 56.1% [10]; Ghana, where the rate is 41.7%; and Nigeria, where the rate is 26.6% [14]. Several factors, such as older age [11,15], being female [10,11,16,17], urban residence [10], being unmarried [17], low education level [11,16], body mass index (BMI) [18], alcohol consumption [17,18], tobacco use [18], duration of hypertension [17], number of medications [18], and comorbidities [11,16,17], were found to be associated with depression among hypertensive patients in African contexts. While depression among hypertensive patients has been studied in such African contexts [10,11,16,17,18], research on patients with hypertension and depressive symptoms has not been conducted in Sudan. This is highly needed to bridge the treatment gap for mental health, especially in low- and middle-income countries [19]. The situation in Sudan could be different from other African countries in terms of sociodemographic characteristics, and these could have an effect on the relationship between hypertension and depression. Additionally, in Sudan, the burden of hypertension is substantial in many regions, including the Eastern Region, with rising prevalence rates attributed to lifestyle changes and socioeconomic factors [6,7,20]. Thus, this study aims to assess the prevalence of depressive symptoms in patients with hypertension in Eastern Sudan and to identify associated factors.

## 2. Materials and Methods

### 2.1. Study Area

This study was conducted in Gadarif Hospital, which is the main hospital in Gadarif State. According to the 2008 census, there are 1,400,000 inhabitants in Gadarif State [21]. Gadarif city is the main city of Gadarif State, located on the Ethiopian border. The inhabitants of Gadarif belong to different Sudanese tribes and work mainly in agriculture [22].

### 2.2. Participants and Study Design

This hospital-based cross-sectional survey was conducted during the period of July to October 2024 at the outpatient internal medicine unit at Gadarif Hospital. Patients (males and females) with hypertension were recruited. The guidelines provided by the Strengthening the Reporting of Observational Studies in Epidemiology (STROBE) initiative were strictly followed [23]. All the methods were performed in accordance with the relevant guidelines and regulations.

### 2.3. Inclusion Criteria

Adult patients with hypertension who were attending follow-up appointments at the clinic were invited to participate in the study. Adult patients with hypertension aged between 30 and 69 years (in Gadarif, patients with hypertension who are under 30 or over 70 years have other specific clinics), able to provide informed consent, and with no physical disabilities or deformities that could interfere with anthropometric assessments were included.

### 2.4. Exclusion Criteria

Participants who were under the age of 30 or who chose not to participate were excluded. Furthermore, individuals were excluded if they had been diagnosed with depression before their hypertension diagnosis.

### 2.5. Sample Size Calculation

The sample size was calculated using the single-population proportion formula. The authors estimated that 30% of patients with hypertension would have depressive symptoms, as there is currently no data on this issue for patients with hypertension in Sudan. This assumption was based on the reported prevalence of depressive symptoms in Ethiopia, which is 32.4% [8]. Additionally, based on another study from Ethiopia, the authors posited that patients with hypertension who used tobacco were at double the risk of experiencing depressive symptoms compared to those who never used tobacco [18]. Consequently, a sample size of 474 hypertensive patients was determined with a 95% confidence level. This sample size would have 80% power, with an alpha level of 0.05, with the expectation that 10% would not respond.

### 2.6. Sampling Procedure

Researchers selected every third consecutive patient with hypertension (starting from the second patient registered in the clinic). If a selected patient was ineligible or declined to participate, the patient immediately following on the list was approached. This sequential replacement process continued until the study reached its target sample size of 474 participants.

### 2.7. Data Collection

Two trained (female and male) medical practitioners used a questionnaire to collect sociodemographic characteristics through face-to-face interviews, including the Patient Health Questionnaire (PHQ-9). The questionnaire was designed according to similar studies conducted in Africa [10,11,16,17,18]. The sociodemographic data collected were age in years, sex, residence (rural or urban), marital status (married, single, divorced, or widowed), education, and occupation. The questionnaire also documented alcohol use (never, current, or former) and tobacco use (never, current, or former. The details on pre-existing conditions/comorbidities—such as diabetes mellitus, ischemic heart disease, and kidney disease—as well as a comprehensive history of hypertension, including how long the patient had hypertension and the medications they were taking, were also gathered. Standardized methods were used to measure each participant’s height and weight, and Body Mass Index (BMI) was calculated as weight in kilograms divided by height in meters squared(kg)/height (m^2^) [24].

The study evaluated depressive symptoms using a validated questionnaire, specifically the Sudanese version of the Arabic translation of the PHQ-9 [25]. The PHQ-9 has nine questions designed to screen for depressive symptoms and to assess their severity. It has demonstrated high sensitivity (73%) and very high specificity (98%) for diagnosing major depression based on structured psychiatric interviews [26]. The PHQ-9 score ranges from 0 to 27, with each symptom rated on a scale from 0 to 3 (0 = Not at all; 1 = Several days; 2 = More than half the days; and 3 = Nearly every day). The prevalence of depressive symptoms was diagnosed using a cut-off score of ≥10, which is the most widely recognized threshold for indicating a positive case of depression [16]. Participants with a score of ≥10 were classified as having depressive symptoms. Patients with severe depressive symptoms were referred to a psychiatrist.

### 2.8. Statistics

Data were analyzed using SPSS for Windows (Version 27.0). the Shapiro–Wilk test was used to assess age, BMI, and all continuous variables for normality, and they were found to be non-normally distributed. Medians (interquartile range, IQR) and proportions were used to express the descriptive statistics. A univariate model was constructed to assess the association between depressive symptoms (dependent variable) and a suite of independent variables: age, sex, residence, BMI, education, occupation, income, tobacco/alcohol history, duration of hypertension, number of medications, and comorbidities. To control for potential confounding variables, predictors with a univariate *p*-value under 0.20 were advanced to the multivariable logistic regression model. Firth’s penalized likelihood logistic regression and bootstrapping were performed to test the sensitivity of the model. Statistical significance was defined as a *p*-value below 0.05, and results are reported using adjusted odds ratios (AORs) alongside their corresponding 95% confidence intervals (CIs).

## 3. Results

### 3.1. General Characteristics

**Figure 1:**
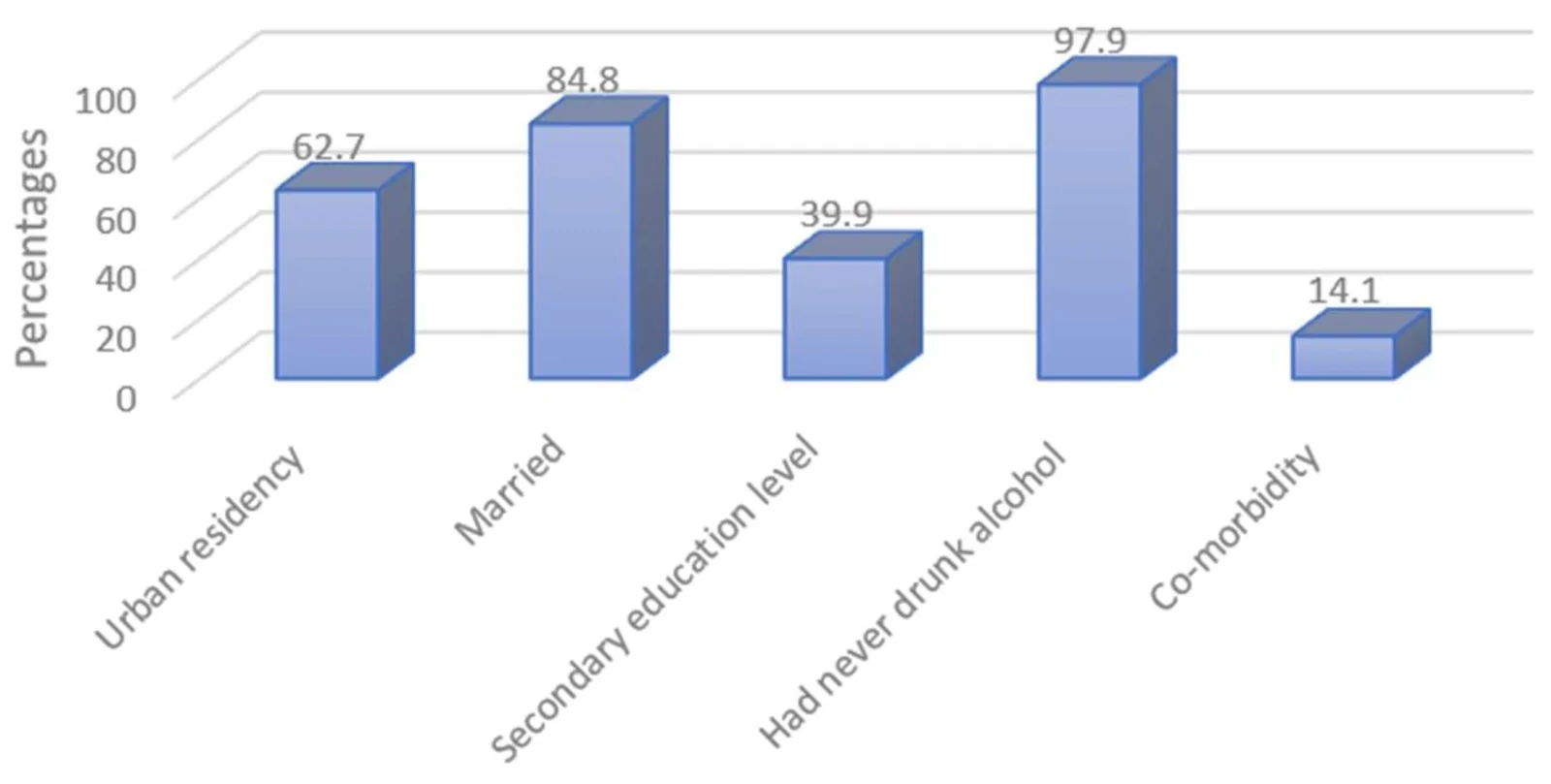
General characteristics of the patients with hypertension in eastern Sudan (n = 474).

Four hundred and ninety-two patients were initially approached to participate. After the exclusion of 18 (3.6) patients, a total of 474 adults with hypertension were enrolled in this study (162 [34.2%] males and 312 [65.8%] females). The median (IQR) age and BMI were 53.0 (45.0–60.0) years and 26.4 (24.2–28.7) kg/m^2^, respectively. The median (IQR) duration of hypertension was 5.0 (3.0–7.0) years. The median (IQR) number of medications was 1 (1–1). Calcium channel blockers (294, 62.0%), angiotensin II receptor blockers (68.0, 14.3%), and angiotensin-converting enzyme inhibitors (35, 7.4%) were the most common drugs used for hypertension. Out of the 474 total participants, 62.7% (n = 297) lived in urban areas, 84.8% (n = 402) were married, and 19.6% (n = 93) were employed. In terms of socioeconomic factors, 39.9% (n = 189) attained at least secondary education, 44.1% (n = 209) reported moderate to high incomes, and 14.1% (n = 67) presented with comorbidities. Regarding lifestyle and health habits, the vast majority had never drunk alcohol (97.9%, n = 464), 11.4% (n = 54) had a history of tobacco use, and 42 (8.9%, 95% CI = 6.5–11.7%) of the adults exhibited depressive symptoms (Figure 1).

### 3.2. Factors Associated with Depressive Symptoms

There was no significant difference in sex, residency, marital status, employment, BMI, income, and types of drugs used for hypertension between hypertensive patients with or without depressive symptoms. The patients with depressive symptoms were significantly older, had a longer duration of hypertension, had an increased number of medications, were educated, had a comorbidity, used tobacco and consumed alcohol (Table 1).

A logistic regression analysis showed that age (AOR = 1.05, 95% CI = 1.01–1.10), being unmarried (AOR = 4.27, 95%, CI 1.71–10.67), and tobacco use (AOR = 2.45, 95% CI = 1.04–5.80) were associated with depressive symptoms. The duration of hypertension, number of medications, education level, and comorbidities were not associated with depressive symptoms. Firth’s penalized likelihood logistic regression and Bootstrap were performed, and the results were not different from the standard maximum likelihood estimates (Table 2). Thus, the dataset did not suffer from small-sample bias or rare-event distortion.

## 4. Discussion

This study assessed the prevalence of depressive symptoms and the factors associated with these symptoms in adult patients with hypertension in Eastern Sudan, and it revealed a noteworthy prevalence rate of 8.9% in Eastern Sudan. Older adults, those who were unmarried, and those who used tobacco were associated with depressive symptoms. The prevalence of depressive symptoms (8.9%) in patients with hypertension was low compared to the prevalence found in other African countries by other studies, such as the prevalence in Ethiopia (32.4%) [8], Morocco (56.1%) [10], Ghana (41.7%), and Nigeria (26.6%) [14]. Notably, a low prevalence of depressive symptoms was reported in the USA, at 12.3% [27]. The low prevalence of depressive symptoms among patients with hypertension in Eastern Sudan compared to other African countries could be attributed to several factors. Cultural perceptions of mental health can influence reporting and diagnosis, potentially leading to an under-recognition of depressive symptoms [28]. Social support systems in some communities may also mitigate the psychological impact of chronic diseases [29]. Access to healthcare services, including mental health resources, might also differ, impacting diagnosis rates [28,29]. Furthermore, variations in lifestyle factors across regions, such as diet, physical activity, and socioeconomic conditions, can contribute to differences in mental health outcomes among hypertensive populations. For example, the high proportion of unmarried participants and the low level of alcohol consumption (97.9 percent never drank) might modify the relationship between hypertension and depression [17,18]. Importantly, various cutoff points for diagnosing depressive symptoms using the PHQ-9 score have been established, ranging from 5 to 27 [14,30] to 10 to 27 [16].

This study revealed that older age was associated with depressive symptoms in patients with hypertension. Other studies have shown similar findings [11,15]. Several interrelated factors can explain this association. First, aging is linked to a higher prevalence of chronic diseases, including hypertension, which can contribute to feelings of helplessness and decreased quality of life [7,29]. The physical limitations imposed by these conditions may lead to social isolation, a known risk factor for depressive symptoms. In addition, older adults may experience significant life changes, such as retirement, the loss of loved ones, or decreased social interactions, that can exacerbate loneliness and depression [31]. Furthermore, the biological changes associated with aging, including alterations in brain chemistry and hormonal changes, can increase vulnerability to mood disorders [32,33]. Research has also shown that cumulative stressors over a lifetime, including healthcare access and socioeconomic challenges, can contribute to mental health decline in older adults with chronic conditions, such as hypertension [15]. Addressing these multifaceted issues is crucial for improving mental health outcomes in this population.

In this study, patients with hypertension who were unmarried (i.e., single, divorced, or widowed) were associated with depressive symptoms. Studies in the neighboring country of Ethiopia found similar results [8,17]. In their systematic review and meta-analysis, Tassew et al. reported that, in Ethiopia, being unmarried put patients with hypertension at twice the risk of depression [8]. Several interconnected factors can explain this association. First, social support plays a crucial role in mental health; those who are unmarried may experience lower levels of emotional and practical support than their married counterparts [34]. This lack of support can exacerbate loneliness and isolation, which are significant risk factors for depressive symptoms. Consequently, poor social support has been found to be associated with depression in patients with hypertension [8,11,16,30]. Second, the stress associated with managing chronic conditions, such as hypertension, can be intensified in unmarried individuals [35]. The absence of a partner to share burdens or provide assistance during health challenges may increase psychological distress. In addition, unmarried individuals, especially divorced women, may face societal stigma or negative perceptions, which can compound feelings of inadequacy [36]. Economic factors may also contribute to this vulnerability. Unmarried adults may have fewer financial resources or face greater economic instability, which can heighten stress levels and negatively impact mental health [37]. Therefore, the combination of social isolation, stress-management challenges, and financial insecurity can significantly increase the risk of depression in unmarried patients with hypertension.

In this study, tobacco-using patients with hypertension were at greater risk of depressive symptoms, which is similar to the findings of other studies [9,18]. There are several reasons for this. First, nicotine and other chemicals in tobacco can alter brain chemistry, affecting neurotransmitter systems, such as dopamine and serotonin, that are critical for regulating moods [38,39]. This alteration can lead to an increased vulnerability to depressive symptoms. Second, the stress and health complications related to hypertension can be compounded by the use of tobacco. Patients with hypertension who smoke may experience more significant physical health challenges, including cardiovascular issues, which can exacerbate anxiety and depression while decreasing the amount of physical activity [40]. The interplay between managing a chronic condition and the adverse health effects of smoking can create a cycle of stress that heightens the risk of depressive symptoms.

Moreover, tobacco use is often associated with social stigma and isolation, which can further contribute to loneliness and despair [41]. Smokers may also face barriers to accessing healthcare resources, including mental health support, which can prevent them from receiving adequate treatment for both hypertension and depressive symptoms. This combination of biological, psychological, and social factors makes tobacco users with hypertension particularly vulnerable to depressive symptoms. The relationship between tobacco use and major depressive disorder is one of the most robust associations in psychiatric epidemiology, with smokers being roughly twice as likely to experience depressive symptoms compared to non-smokers. To understand why this overlap exists, researchers look at the arrow of causality. While traditional models long assumed that tobacco use causes or exacerbates mental health struggles, modern research emphasizes bidirectionality, reverse causality, and the self-medication hypothesis [42].

In contrast to this study, other studies found that being female [10,11,16,17], an urban residence [10], a low education level [11,16], BMI [18], the duration of hypertension [17], the number of medications [18], the type of drug used to treat hypertension [43], and comorbidities [11,16,17] were associated with depressive symptoms in patients with hypertension in various African contexts. Researchers should view these contradictory results as an opportunity to evaluate their own country or region and to develop locally based solutions.

### 4.1. Strengths and Implications of This Study

The strengths of this study include the following:

Focused Population: This study targets the population of patients with hypertension in Eastern Sudan, which allows for a detailed understanding of the prevalence of depressive symptoms in this group. This focus can lead to tailored interventions.

Hospital-Based Data Collection: Conducting the study in a hospital setting ensures access to relevant patient demographics, which enhances the reliability of the collected data on hypertension and depression.

Cross-Sectional Design: The cross-sectional design supports an assessment of the prevalence of depressive symptoms at a single point in time, providing a clear snapshot that can inform healthcare providers about the immediate mental health needs of patients with hypertension.

The implications of this study are as follows:

Healthcare Policy: The findings can inform healthcare policymakers in Sudan about the importance of integrating mental health services into the management of chronic conditions, such as hypertension, which can potentially lead to improved patient outcomes.

Awareness and Training: The results highlight the need for increased awareness and training for healthcare providers regarding the mental health aspects of managing hypertensive patients, which can foster a more holistic approach to treatment.

Further Research Directions: This study can pave the way for future research on the interplay between hypertension and mental health, and it should encourage longitudinal studies that explore the long-term effects of depression on hypertension management and the causal relations between them.

Overall, this study contributes significantly to the understanding of mental health in the context of chronic disease and low-resource settings.

### 4.2. Limitations of This Study

Despite the strengths mentioned above, this study has limitations that must be acknowledged to improve future research designs. These limitations included the following: Cross-Sectional Nature: The study’s cross-sectional design prevents the determination of causal relationships between hypertension and depression. While associations can be observed, it is unclear whether hypertension contributes to the development of depression or if pre-existing depression influences hypertension. Kariis et al. reported that depression is associated with lower persistence and adherence to antihypertensive medication [13]. Limited Geographic Scope: Conducting the study in a single hospital in Eastern Sudan may limit the sample’s representativeness. The findings may not be generalizable to hypertensive patients in other Sudanese regions or to those receiving care in different healthcare settings. Potential Response Bias: Using self-reported questionnaires to assess depression and associated factors can lead to response bias. Participants might underreport symptoms due to the stigma surrounding mental health issues, or they might misinterpret the questions, which can reduce the accuracy of the data collected. Moreover, the low events-per-variable ratio and the risk of overfitting; the lack of a Sudanese validation study for the PHQ-9; the exclusion of patients with pre-existing depression, which may underestimate prevalence; the absence of data on social support, which the discussion identifies as a key mechanism; and the potential for selection bias due to the hospital-based sampling frame are limitations of the study.

## 5. Conclusions

This study reveals that among the hypertensive adults in the sample, 8.9% experienced depression, and this was significantly associated with older age, being unmarried, and tobacco use. There is a need to introduce routine PHQ-9 screening in this setting. These findings emphasize the need for targeted mental health interventions that address social and lifestyle factors to improve the overall well-being of hypertensive patients.

## Figures and Tables

**Table 1:** Univariate analysis for factors associated with depressive symptoms among patients with hypertension in Eastern Sudan, 2024 (n = 474).

Variable		Depressive Symptoms		Odds Ratio	95% Confidence Interval	*p* Value
		Yes (n = 42)	No (n = 432)			
		Median (interquartile range)				
Age, years		60.5 (50.2–68.2)	52.0 (45.0–59.0)	1.07	1.03–1.10	<0.001
Body mass index, kg/m2		27.4 (23.5–29.3)	26.2 (24.2–28.7)	1.04	0.96–1.12	0.307
Duration of hypertension		6.0 (3.0–10.0)	5.0 (3.0–7.0)	1.09	1.03–1.14	0.001
Number of medications		2.5 (1.0–4.0)	1.0 (1.0–1.0)	1.63	1.34–1.97	<0.001
		Frequency (percentage)				
Sex	Male	16 (38.1)	146 (33.8)	Reference		
	Female	26 (61.9)	286 (66.2)	0.83	0.43–1.59	0.575
Marital status	Married	32 (76.2)	370 (85.6)	Reference		
	Unmarried	10 (23.8)	62 (14.4)	1.86	0.87–3.98	0.108
Residency	Rural	15 (35.7)	162 (37.5)	Reference		
	Urban	27 (64.3)	270 (62.5)	1.08	0.55–2.09	0.819
Education status	≥Secondary	9 (21.4)	180 (41.7)	Reference		
	<Secondary	33 (78.6)	252 (58.3)	2.62	1.22–5.60	0.013
Occupation status	Unemployed	35 (83.3)	346 (80.1)	Reference		
	Employed	7 (16.7)	86 (19.9)	0.80	0.34–1.87	0.614
Comorbidity	No	24 (57.1)	383 (88.7)	Reference		
	Yes	18 (42.9)	49 (11.3)	5.86	2.97–11.56	<0.001
Tobacco use	Never	29 (69.0)	391 (90.5)	Reference		
	Current/former	13 (31.0)	41 (9.5)	4.27	2.06–8.86	<0.001
Alcohol consumption	Never	39 (92.9)	425 (98.4)	Reference		
	Current/former	3 (7.1)	7 (1.6)	4.67	1.16–18.78	0.030
Income	High/moderate	27 (64.3)	238 (55.1)	Reference		
	Low	15 (35.7)	194 (44.9)	0.68	0.35–1.31	0.254
Types of antihypertensive	Calcium channel blockers	22 (52.4)	272 (63.0)	Reference		
	Others	20 (47.6)	160 (37.0)	1.54	0.81–2.92	0.280

**Table 2:** Multivariate analysis for factors associated with depressive symptoms among patients with hypertension in Eastern Sudan, 2024 (n = 474).

Variable		AOR	95% CI	*p* Value	Firth AOR (95% CI)	Bootstrap 95% CI	Same Direction, %
Age, years		1.05	1.01–1.10	0.045	1.05 (1.01–1.10)	1.01–1.11	99
Duration of hypertension		1.01	0.96–1.06	0.668	0.98 (0.90–1.06)	0.89–1.06	70
Number of medications		1.20	0.88–1.62	0.244	1.24 (0.91–1.67)	0.89–1.77	89
Marital status	Married	Reference					
	Unmarried	4.27	1.71–10.67	0.002	4.07 (1.64–9.88)	1.47–11.80	99
Education status	≥Secondary	Reference	0.66				
	<Secondary	1.53	0.66–3.57	0.322	1.51 (0.68–3.58)	0.75–4.40	88
Comorbidity	No	Reference					
	Yes	1.84	0.61–5.57	0.276	1.78 (0.60–5.19)	0.50–5.98	85
Tobacco use	Never	Reference					
	Current/former	2.45	1.04–5.80	0.040	2.40 (1.00–5.46)	0.82–5.94	96

Bootstrap: 2000 samples, percentile intervals. Same direction: share of bootstrap samples in which the AOR fell on the same side of 1 as the main estimate. Calibration slope after bootstrap correction: 0.86. C statistic: 0.76. AOR, adjusted odds ratio. CI, confidence interval.

## Data Availability

The data presented in this study are available on request from the corresponding author due to privacy.
